# 2022 ETA Consensus Statement: What are the indications for post-surgical radioiodine therapy in differentiated thyroid cancer?

**DOI:** 10.1530/ETJ-21-0046

**Published:** 2021-10-04

**Authors:** Furio Pacini, Dagmar Fuhrer, Rossella Elisei, Daria Handkiewicz-Junak, Sophie Leboulleux, Markus Luster, Martin Schlumberger, Johannes W Smit

**Affiliations:** 1 Section of Endocrinology, University of Siena, Siena, Italy; 2 Department of Endocrinology, Diabetes and Metabolism, West German Cancer Centre (WTZ), University Hospital Essen, University Duisburg-Essen, Essen, Germany; 3 Section of Endocrinology, Department of Clinical and Experimental Medicine, University of Pisa, Pisa, Italy; 4 Department of Nuclear Medicine and Endocrine Oncology, Maria Skłodowska-Curie National Research Institute of Oncology, Gliwice Branch, Gliwice, Poland; 5 Gustave Roussy Cancer Campus and University Paris-Saclay, Villejuif, Cedex, France; 6 Department of Nuclear Medicine, University Hospital Marburg, Marburg, Germany; 7 Radboud University Medical Center, Nijmegen, Netherlands

**Keywords:** differentiated thyroid cancer, radioiodine, treatment, indications

## Abstract

Modern use of post-operative radioactive iodine (RAI) treatment for differentiated thyroid cancer (DTC) should be implemented in line with patients’ risk stratification. Although beneficial effects of radioiodine are undisputed in high-risk patients, controversy remains in intermediate-risk and some low-risk patients. Since the last consensus on post-surgical use of RAI in DTC patients, new retrospective data and results of prospective randomized trials have been published, which have allowed the development of a new European Thyroid Association (ETA) statement for the indications of post-surgical RAI therapy in DTC. Questions about which patients are candidates for RAI therapy, which activities of RAI can be used, and which modalities of pre-treatment patient preparation should be used are addressed in the present guidelines.

## Introduction

Differentiated thyroid cancer (DTC) accounts for more than 90% of all thyroid cancers. Over the last decades, an increasing incidence of DTC, mainly due to cancers of papillary histology, has been reported in many countries in- and outside of Europe (1, 2). This increase is largely attributable to a better detection of small papillary thyroid carcinomas (PTC), as a result of screening bias (non-selective use of neck ultrasound and fine needle aspiration cytology) (3). Thus, about 70–80% of thyroid carcinomas detected nowadays have PTC with an excellent long-term prognosis for whom overtreatment should be avoided.

Unfortunately, for many years the management of thyroid cancer has been based on retrospective studies which may be biased in many respects. Nowadays, whenever available we should rely on prospective studies which are feasible, as demonstrated by several trials (4, 5, 6, 7).

### Definition of RAI therapy

After total thyroidectomy, radioactive iodine (RAI) therapy can be administered to patients with DTC for various indications.

The non-descript colloquial use of the word 'ablation' has thus far frustrated a constructive scientific dialogue. While it has generally been recognized that the first administered activity of RAI after thyroidectomy can be used in attempts to destroy (first) presumably benign residual thyroid tissue, (second) suspected but not identified remaining disease, and/or (third) known residual or recurrent disease; a precise nomenclature to describe these three important goals has not been widely accepted (8). In a common proposal between the European Thyroid Association (ETA), American Thyroid Association (ATA), European Association of Nuclear Medicine (EANM) and Society of Nuclear Medicine and Molecular Imaging (SNMMI), it is suggested to adopt a nomenclature that uses 'RAI therapy' as the broad term that encompasses the three primary goals associated with an administered activity of RAI: (a) remnant ablation, (b) adjuvant treatment or (c) treatment of known disease (9).

In this context, remnant ablation refers to the use of RAI to destroy post-operatively remaining, presumably benign residual thyroid tissue to facilitate follow-up studies (such as serum thyroglobulin and RAI imaging).

Within the context of thyroid cancer care, adjuvant therapies can be defined as I-131 therapy after surgical resection of all known primary tumor tissue and metastatic foci in an effort to destroy subclinical microscopic tumor deposits that may or may not be present. The goals of adjuvant therapy are to improve disease-specific survival and disease-free survival (9).

Treatment of known disease refers to the goal of destroying persistent or recurrent DTC foci with RAI in order to improve progression free, disease-specific and overall survival. It can be given either with curative or palliative intent (9).

After total thyroidectomy, the size of normal, presumably benign remnants is usually small, resulting in very low or frequently even undetectable serum thyroglobulin (Tg) levels (at least in the hands of large volume surgeons) on levothyroxine (LT4) treatment. In these cases, particularly in low-risk patients, the goal of remnant ablation is already achieved following the surgical procedure alone just by the surgical procedure, and thus, there is no rationale to perform RAI ablation (10, 11). Currently, even without post-operative RAI therapy most patients can be followed up with serum Tg on l-T4 treatment: an undetectable level is reassuring as well as a low but detectable level. In the latter case, the trend of serum Tg over time should be monitored: a declining or a stable Tg is reassuring, whereas an increase should lead to imaging in order to localize and treat the disease and possibly RAI therapy (8).

It is apparent that remnant ablation is aimed to simplify follow-up in any patient regardless of his/her specific risk, while adjuvant treatment is aimed to reduce disease recurrence and cause-specific mortality (10). Nowadays, the large majority of thyroid cancer patients have a low risk of recurrence after complete surgery and an even smaller risk of thyroid cancer related death, and careful examinations of patients’ outcome suggests that the use of post-surgical RAI ablation may be tailored according to a risk-based approach. This is even more relevant in view of changes in DTC management, preceding a decision on RAI therapy, taking a de-escalating approach with increasing use conservative DTC surgery (lobectomy rather than total thyroidectomy), changes in DTC nomenclature, for example, NIFTP and the concept of 'active surveillance' in (very) low-risk PTC (8, 12).

Indeed, despite the inevitable body radiation exposure, the risk of the administration of a low activity of RAI has not been demonstrated in terms of secondary cancer or leukemia, infertility and untoward pregnancy outcomes or other side effects, but, as this is the rule for any treatment modality, expected benefits are warranted to justify its administration (13, 14). In the field of oncology benefits are defined as improvement of overall survival or disease-free survival and quality of life (15).

This European Thyroid Association (ETA) Consensus Statement aims to deliver rational recommendations for the indications of post-operative RAI therapy with its various goals. In particular, we have addressed the issues of which patients are candidates for which form of RAI therapy, which activities of RAI can be used in each scenario and which modalities of preparation should be used. However, this Consensus Statement is mostly based on retrospective studies and biases cannot be excluded, in particular in the selection of patients. In fact, the only way to scientifically compare two treatment modalities and to exclude biases is to perform randomized prospective studies, as such studies are clearly feasible, as already mentioned (4, 5, 6, 7).

## Risk stratification to assess the need for post-operative RAI administration

**Recommendation 1: The decision for post-operative RAI therapy should be taken based on initial prognostic indicators for thyroid cancer related death and recurrence, including among others the surgical and pathological report and on the results of serum Tg measurements and neck ultrasonography obtained after surgery.**

The expected benefit of post-operative RAI therapy depends on the individual risk of the patient (Table 1).

**Table 1:** Tabulated summary of the ETA Consensus Statement.

Recommendation	Factors to be considered
RAI therapy should be based on initial prognostic indicators for thyroid cancer-related death and recurrence	- ATA risk groups: (1) low; (2) intermediate; (3) high- Post-surgical evaluation: (1) neck ultrasound; (2) thyroglobulin
The use of I-131 therapy as adjuvant treatment or treatment of known disease is indicated in the high-risk group	- Overall survival and disease-free survival are improved with RAI- Activities >3700 MBq should be considered
In the intermediate-risk category, RAI therapy may be indicated according to individual risk factors	- The greatest benefit in patients with: (1) advanced age; (2) aggressive histologies; (3) increasing volume of nodal disease; (4) extranodal extension of the tumour; (5) multiple N1; (6) and/or lymph node metastases outside the central neck- Final results of prospective trials expected
In low-risk patients, RAI therapy should be based on individual risk modifiers	- RAI treatment not indicated in PTC < 1 cm (uni- or multifocal)- Abnormal neck ultrasound or high Tg may indicate need for RAI therapy
Recombinant human TSH is preferred for TSH stimulation	- Indicated for all RAI activities- Approved in all risk groups, but metastatic disease
Activities of 1110 MBq are equally effective as higher activities for remnant ablation	- If low-risk patients are referred for thyroid remnant ablation, activity of 1110 MBq should be considered as effective and safer than higher activities
Before RAI therapy diagnostic scan is not routinely required	- RAI low activities before RAI treatment can induce stunning and reduce treatment effectiveness
Before RAI therapy any iodine-containing drug should be avoided	- Low-iodine diet may be advised

The 8th edition of the TNM classification individualizes patients at low risk of thyroid cancer-related death (<2%) who represent the large majority of DTC patients and a small subgroup of patients (5–10%) for whom the risk is higher (16).

The risk of persistent or recurrent disease is indeed higher than the risk of cancer related death, and the ATA has defined three groups of patients with different risk of recurrence (8):

***ATA high risk (>20%) category*** includes patients with: (i) macroscopic invasion of tumor into the perithyroidal soft tissues; (ii) incomplete tumor resection; (iii) distant metastases; (iv) post-operative serum Tg suggestive of distant metastases; (v) pathologic N1 with any metastatic lymph node ≥3 cm in largest dimension; and (vi) follicular thyroid cancer with extensive vascular invasion (>4 foci of vascular invasion). These patients also have a high risk of cancer related death.
***ATA intermediate risk (5–20%) category*** includes patients with: (i) microscopic invasion of tumor into the perithyroidal soft tissues; (ii) aggressive histology (e.g. tall cell, hobnail variant, columnar cell carcinoma); (iii) PTC with vascular invasion; (iv) clinical N1 or >5 pathologic N1 with all N1 <3 cm in largest dimension; (v) multifocal papillary microcarcinoma with microscopic invasion of tumor into the perithyroidal soft tissues and BRAFV600E mutation (if known); tumour larger than 1 cm with BRAF v600E mutation could confer an intermediate risk of recurrence but has not been proven yet, based on a prospective study.
***ATA low risk (<5%) category*** is defined as: (i) intrathyroidal PTC without vascular invasion, with or without small volume lymph node metastases (clinical N0 or ≤5 pathologic N1, all <0.2 cm in largest dimension); (ii) intrathyroidal encapsulated follicular variant of papillary thyroid cancer or intrathyroidal well-differentiated follicular cancer with capsular or minor vascular invasion (<4 vessels involved); (iii) intrathyroidal papillary microcarcinomas that are either BRAF WT or BRAF mutated (if known). Finally, minimal extrathyroidal extension appears to have little impact on outcome and in the 2017 TNM classification for the risk of thyroid cancer related death is no longer taken into account. Several studies have found no difference in recurrence-free survival between patients with or without minimal extrathyroidal extension and the administration of RAI was not related to survival or recurrence (17, 18, 19).

It is important to note that, regardless of the risk category, the results of post-surgical neck ultrasound performed in the immediate post-operative setting (i.e. 2 weeks to 2 months) and serum Tg measurement obtained at 2 weeks to 2 months (preferably more than 6 weeks) or more after surgery have a pivotal role in patient selection for RAI therapy (20, 21, 22, 23). Evidence of biopsy proven lymph node metastases and/or unstimulated post-operative Tg values above an institutional cut-off (e.g. >2 ng/mL) should lead to selection for RAI.

Whenever risk factors and patients’ selection for total thyroidectomy and radioiodine are evaluated, wide differences between countries as environmental factors, preclinical care and healthcare should also be considered.

**Recommendation 2: The use of I-131 therapy as adjuvant treatment or treatment of known disease is indicated for patients in the high risk of recurrence category or with known structural disease. In this setting, high activities (≥3700 MBq)of radioiodine are preferred over low activities.**

This recommendation is based on the evidence from retrospective or non-controlled prospective studies that both overall survival and disease-free survival, are improved with post-operative radioiodine administration (24). In this situation, RAI administration is clearly intended more as an adjuvant treatment or treatment of known disease rather than for thyroid remnant ablation.

For activity selection, the precise goal needs to be considered. There is no evidence that for adjuvant treatment, more than 3700 MBq will further improve prognosis. For treatment of known disease, there are differing opinions in literature on whether standard activities >3700 MBq or dosimetrically determined activities are of benefit. However, the only large comparative series available (25) does not show any benefit of either dosimetry or activities >3700 MBq.

**Recommendation 3: In the intermediate-risk category, RAI therapy may be indicated and should be tailored according to individual cases.**

The greatest benefit of post-operative I-131 therapy may be expected in patients with advanced age, with aggressive histologies, increasing volume of nodal disease, extranodal extension of the tumor, multiple N1 and/or lymph node metastases outside the central neck. In these patients, RAI therapy may be given as an adjuvant treatment.

In patients with minimal extrathyroidal invasion, microscopic or few lymph node metastases and intrathyroidal PTC with BRAFV600E mutation, RAI therapy can be decided based on post-operative Tg and neck ultrasound. Indeed, in this category of patients the benefit of RAI therapy is controversial (26, 27). In addition, we should consider that elderly patients, those with an aggressive histology and those with BRAF mutations frequently do not concentrate radioiodine (28, 29).

Aggressive histologies, such as tall cell variant, columnar and hobnail variant have a lowered likelihood of RAI uptake, and BRAF mutations are found with higher prevalence in these tumors than in classical PTC. As already mentioned, many tumors with aggressive histology, do not express NIS and do not have RAI uptake. This is the basis for redifferentiation protocols and such patients should be preferably offered to enter clinical trials, rather than treated blindly.

Sacks *et al*. (30) found that cause-specific survival was improved in patients aged >45 years with primary tumors >4 cm, microscopic extrathyroidal invasion, and/or lymph node metastases (UICC/AJCC TNM stage III). It is not clear whether younger patients with lymph node metastases benefit similarly from RAI therapy. In their meta-analysis, Sacks *et al*. (30) concluded that post-operative I-131 administration did not improve survival or recurrence in patients aged <45 years with microscopic central compartment lymph node metastases, whereas a benefit was uncertain in the setting of lateral or macroscopic lymph node metastases. Aggressive variants of PTC, such as diffuse sclerosing (DSV) and tall cell (TCV) variants, were associated with a reduced overall survival in intermediate-risk PTC patients. Patients with DSV and TCV who did not receive RAI were 4.9 and 2.1 times more likely to die compared to patients who received RAI (31, 32). In addition, in another study after exclusion of aggressive variants, overall survival was better in intermediate-risk patients with lymph node metastases and/or extrathyroidal invasion treated with RAI (33). RAI was associated with a 29% reduction in the risk of death, including patients younger than 45 years (36% reduction in risk of death). The absolute risk difference for overall survival would be estimated at 1% in younger (<45 years) and 4% in older (≥65 years) patients, respectively. At variance (34) both children and adults with MACIS <6 PTC have a <1% chance at 30 years of cause specific mortality.

**Recommendation 4: In low-risk patients, the benefit of I-131 therapy is a matter of intensive scientific debate and the decision on whether to perform RAI therapy should be based on the presence of individual risk modifiers.**

There is controversial discussion among experts on whether post-operative I-131 administration is useful in low-risk patients. In absolute terms, in these patients the risk of disease-specific deaths is less than 1% and that of persistent/recurrent disease is low (2–3%). Thus, the challenge is to identify those patients who should be treated with RAI and to avoid therapy in patients, who will potentially not benefit from the procedure. It is hard to imagine that retrospective studies can demonstrate any benefit in terms of disease-free survival when the overall risk is as low as 2–3%. As summarized in a meta-analyses by Sawka *et al*. in 2008 (28) with an update by Verburg in 2020 based on the literature of the past decade (35), the results are not consistent. Some authors report a benefit of applying RAI even to patients with non-metastasized microcarcinomas, whereas other groups find no benefit at all. A tendency for larger groups and longer follow-up duration seems to be loosely associated with showing an advantage of giving RAI – but not consistently across the available reports. Although all authors are studying the same disease and the same therapeutic modality, the variability of the results shows that likely large and important sources of heterogeneity in outcome have thus far neither been identified nor studied sufficiently. An exception to this uncertainty concerns patients with abnormal ultrasound and/or elevated Tg levels (24, 36, 37, 38, 39).

RAI remnant ablation is unlikely to improve the outcome of papillary microcarcinoma (<1 cm, uni- or multi-focal), in absence of other higher-risk features (40, 41) and RAI should not be used in these patients. However, low-risk patients with post-operatively detectable serum Tg, in particular when it is above the institutional cut-off of, for example, 2 ng/mL on l-T4 or >5–10 ng/mL after TSH stimulation or with abnormal ultrasound findings have a higher risk of recurrence, and RAI therapy may be considered, although there is no evidence that it can improve disease-free survival. Currently, two major randomized trials comparing the outcome of low-risk patients receiving post-operative RAI therapy vs no RAI are ongoing in France (ESTIMABL 2) and UK (Ion). The ESTIMABL 2 study randomized 776 low-risk DTC patients to a follow up without adjuvant RAI or to adjuvant treatment with 1110 MBq of RAI after rhTSH stimulation. First results showed that 3 years after randomization the rate of patients without events defined by new RAI administration, surgery or biological abnormalities were similar in both groups (42). Results of the study indicate that there is no need for remnant ablation in this group of DTC patients.

In contrast, the risk of persistent disease is approximately 1.5% when serum Tg is undetectable on l-T4 treatment or <1 ng/mL after TSH stimulation. These patients will potentially not benefit from RAI ablation. In addition, even if no I-131 therapy be given after surgery, clinical recurrences can be treated successfully later on.

## Preparation for RAI administration

**Recommendation 5: Recombinant human TSH during l-T4 treatment should be the preferred method of preparation for RAI administration.**

Remnant ablation has historically been performed after prolonged l-T4 withdrawal to increase endogenous thyroid-stimulating hormone (TSH) to levels sufficient to induce robust RAI uptake in thyroid cells. However, this induces hypothyroidism with a major decrease in quality of life, which may last for up to 2–3 months. Empirically, it is estimated that a TSH >30 mU/L is a good cut-off (43), but no comparative study has ever validated this assumption and more recent results seem to contradict this assumption (44). For thyroid hormone withdrawal, two possible approaches are used: (i) switch from l-T4 to triiodothyronine (L-T3) for 2–3 weeks (3, 4) and then stop l-T3 for 2 weeks, or (ii) stop L-T4 for 3–4 weeks without switching to l-T3. Either method will significantly decrease quality of life. Alternatively, induction of a less prominent hypothyroidism (reducing L-T4 half the original dose) has also been proposed (45).

For nearly two decades now, a second method of preparation for RAI therapy has been available: the i.m. administration of exogenous recombinant human TSH (rhTSH) 0.9 mg on 2 consecutive days with RAI administration on the day following the second rhTSH injection. A prospective, multicenter, randomized study demonstrated that remnant ablation with 3700 MBq is equally effective after either rhTSH stimulation or prolonged thyroid hormone withdrawal (46). In another study, remnant ablation rates using 1850 MBq of I-131 were similar with either withdrawal or preparation with rhTSH (47). Recently, two randomized non-inferiority trials comparing low and high activities of radioiodine, each in combination with either rhTSH or thyroid hormone withdrawal (THW), have been published (4, 5). The majority of patients were 'low risk' but some patients at 'intermediate risk' (with lymph node metastases or minimal extrathyroidal invasion) were also included in the British HiLo study (5). The remnant ablation rate was similar in all groups irrespective of the TSH stimulation method used, and the authors concluded that recombinant human thyrotropin is equally effective for preparation for remnant ablation in low-risk patients. In addition, short-term recurrence rates have been found to be similar in patients prepared with thyroid hormone withdrawal or rhTSH, both in low (11, 48) and intermediate-risk patients (49, 50). Furthermore the preparation with rhTSH is associated with an unimpaired quality of life (46, 51), and reduces both the whole body radiation absorbed dose (38, 39) and duration of hospitalization (6, 41, 43, 52). As the use of rhTSH is approved for 'ablation', with any RAI activity, both in the United States and Europe, for any patient except those with distant metastases, it is advocated to use rhTSH as the preferred method of patient preparation for I-131 administration in patients that fall within the registration label.

## Activity of I-131 to be employed for post-surgical thyroid remnant ablation

**Recommendation 6: Activities of 1110 MBq are equally effective as higher activities for ablation of presumably benign thyroid remnants.**

Although there is a trend toward higher ablation rates with higher activities in patients with large thyroid remnants as observed in the past, similar rates of successful remnant ablation have been reported using activities ranging from 1110 to 3700 MBq of I-131 (47, 48, 49, 50). A randomized study using preparation with rhTSH showed that ablation rates were comparable with 1850 MBq or 3700 MBq (47). In another prospective, randomized trial in 160 patients, comparing ablation with 1110 MBq and 3700 MBq, the authors found no difference in the ablation rate between activities (49). The two prospective randomized studies in France and England referred to before found no signiﬁcant difference in the remnant ablation rate using 1110 MBq or 3700 MBq of I-131, either after preparation with thyroid hormone withdrawal or rhTSH (4, 5).

Concerning the issue of the follow up of patients treated with a low activity of I-131, a prospective, randomized study comparing the rate of recurrent disease in low-risk patients ablated with 1110 MBq or 3700 MBq, showed that the rate of persistent disease was similar in both groups ovrer a 10-year follow-up (49). In contrast, a higher DTC-related mortality was recently reported in low- and high-risk patients treated with low activities at initial I-131 therapy (≤2000 MBq) when patients were at least 45 years of age at diagnosis, as well as a higher recurrence rate in older high-risk patients without distant metastases (51).

## Should diagnostic RAI scanning be performed before RAI therapy?

**Recommendation 7: Whenever a decision to perform post-operative RAI therapy needs to be taken, a diagnostic scan is not routinely required.**

A diagnostic RAI whole body scan (WBS) provides information on the presence of iodine-avid thyroid tissue, both normal and malignant. There is an increasing trend to avoid diagnostic RAI WBS before post-operative I-131 therapy because of its low impact on the decision to treat, and because of concerns regarding I-131-induced stunning of thyroid remnants (53, 54) and metastases (55). The alternative radiopharmaceuticals for staging, I-123 or I-124, are not readily or cheaply available (56).

It is recommended to perform a post-therapy WBS within one week after the administration of RAI. This imaging technique is of paramount importance in confirming the presence and the extent of the thyroid remnant and may disclose the presence of unsuspected metastatic foci in 10–26% of high-risk cases (57) and more rarely also in low-intermediate-risk patients, thus allowing the re-classification of disease stage (58). Whenever possible, a single photon emission computed tomography (SPECT)/computed tomography (CT) is to be performed to better define the neck uptake and to distinguish the thyroid remnants from locoregional lymph node metastases (59).

## Is a low-iodine diet necessary before RAI administration?

**Recommendation 8: A low-iodine diet may be prescribed but its utility is not demonstrated unequivocally. Any iodine-containing drug should be avoided.**

Exposure to excessive amounts of stable iodine may influence the uptake of diagnostic or therapeutic activities of RAI. Several centers advocate preparation of the patients with a low-iodine diet (LID) and recommend avoiding excessive iodine exposure (i.v. contrast agent, amiodarone or any other iodine-containing drugs) prior to RAI therapy. However, no prospective study has ever determined the cut-off over which interference may actually occur.

In a recent systematic review, a LID allowing for ≤50 µg/day of iodine for 1–2 weeks prior to RAI administration appeared to be associated with an increase in RAI uptake, compared to no LID (60), but there is conflicting evidence on the impact of LID on the remnant ablation success. In a retrospective study, aimed to compare different levels of urinary iodine excretion on the results of thyroid ablation in patients not prepared with low iodine diet, the authors found no influence of the levels of urinary iodine on the outcome of thyroid ablation up to urinary iodine levels of 350 µg/day (61). In another study (62), a low-iodine diet was associated with a higher rate of remnant ablation. Measurement of urinary iodine excretion (when available) before RAI therapy may help in detecting the few cases with a significant iodine excess (63).

## Conclusions

Careful analysis of patients’ outcome has introduced the concept or risk-based selection of candidates for post-operative I-131 therapy. In accordance with this concept, RAI therapy is recommended based on the individual risk assessment.

## Declaration of interest

Daria Handkiewicz-Junak: travel grants from Genzyme-Sanofis. Sophie Leboulleux: Advisory Board membership for Bayer, Lilly and EISAI. Furio Pacini, Dagmar Fuhrer, Rossella Elisei, Markus Luster, Martin Schlumberger and Jan Smit declare that there is no conflict of interest that could be perceived as prejudicing the impartiality of this work.

## Funding

This work did not receive any specific grant from any funding agency in the public, commercial or not-for-profit sector.
